# Novel molecular mechanisms in Alzheimer’s disease: The potential role of DEK in disease pathogenesis

**DOI:** 10.3389/fnagi.2022.1018180

**Published:** 2022-10-06

**Authors:** Allie N. Greene, Matia B. Solomon, Lisa M. Privette Vinnedge

**Affiliations:** ^1^Neuroscience Graduate Program, University of Cincinnati College of Medicine, Cincinnati, OH, United States; ^2^Department of Psychology, University of Cincinnati, Cincinnati, OH, United States; ^3^Division of Oncology, Cancer and Blood Diseases Institute, Cincinnati Children’s Hospital Medical Center, Cincinnati, OH, United States; ^4^Department of Pediatrics, University of Cincinnati College of Medicine, Cincinnati, OH, United States

**Keywords:** DEK, Tau, dementia, neurodegenerative disease, Alzheimer’s disease

## Abstract

Alzheimer’s disease and age-related dementias (AD/ADRD) are debilitating diseases that exact a significant physical, emotional, cognitive, and financial toll on the individual and their social network. While genetic risk factors for early-onset AD have been identified, the molecular and genetic drivers of late-onset AD, the most common subtype, remain a mystery. Current treatment options are limited for the 35 million people in the United States with AD/ADRD. Thus, it is critically important to identify novel molecular mechanisms of dementia-related pathology that may be targets for the development of new interventions. Here, we summarize the overarching concepts regarding AD/ADRD pathogenesis. Then, we highlight one potential molecular driver of AD/ADRD, the chromatin remodeling protein DEK. We discuss *in vitro*, *in vivo*, and *ex vivo* findings, from our group and others, that link DEK loss with the cellular, molecular, and behavioral signatures of AD/ADRD. These include associations between DEK loss and cellular and molecular hallmarks of AD/ADRD, including apoptosis, Tau expression, and Tau hyperphosphorylation. We also briefly discuss work that suggests sex-specific differences in the role of DEK in AD/ADRD pathogenesis. Finally, we discuss future directions for exploiting the DEK protein as a novel player and potential therapeutic target for the treatment of AD/ADRD.

## Epidemiology

Alzheimer’s disease (AD), the most common cause of dementia, currently affects 50 million people worldwide, which is 5% of the world’s elderly population (World Health Organization [WHO], 2017). This number could more than double by the year 2050 (World Health Organization [WHO], 2017). The pathogenesis of Alzheimer’s disease has been an enigma to clinicians, researchers, caretakers, and patients for over a century. For example, the onset of cellular and molecular pathology in the brain can precede clinical symptoms for years, or even decades (DeTure and Dickson, 2019). Therefore, it is exceedingly difficult to diagnose AD early enough to prevent progression of the disease when the irreversible molecular hallmarks of brain pathology, i.e., the aggregation of amyloid beta (Aβ) plaques and neurofibrillary Tau tangles, have become pronounced. AD is predominantly diagnosed through cognitive assessments and by subsequently ruling out other causes of dementia (Apostolova, 2016). The main symptoms present when AD is diagnosed are memory loss, confusion, and difficulties thinking and communicating (Alzheimer’s Association, 2019a). As the disease progresses, patients may even experience changes in personality and behavior, such as anxiety, agitation, or delusions (Alzheimer’s Association, 2019a). The diagnosis of other dementia types such as vascular dementia, Huntington’s disease, Lewy body dementia, or Parkinson’s disease, also relies heavily on assessment of behavioral symptoms, such as motor impairment or language difficulties. Detection of brain-based molecular changes in dementias are most efficiently diagnosed post-mortem, such as the aggregation of alpha-synuclein in Parkinson’s disease, but trials of biomarker detection in various neurodegenerative disorders are underway to improve dementia diagnoses.

There are two major types of AD. One, called early-onset AD, occurs when a patient experiences the onset of dementia symptoms and receives an AD diagnosis before the age of 65 (World Health Organization [WHO], 2017). Worldwide, early-onset AD accounts for 5–10% of cases (World Health Organization [WHO], 2017). The main cause of early-onset AD is one’s genetic predisposition facilitated by familial inheritance of mutations in specific genes: amyloid precursor protein (APP), presenilin 1, and presenilin 2 (Wu et al., 2012). Mutations in these genes lead to pathogenic processing of APP to increase expression of Aβ 42 (Dai et al., 2017), a 42-residue-long fragment of APP which aggregates into Amyloid plaques in the brains of AD patients (Burdick et al., 1992; Gravina et al., 1995; Sun et al., 2015). In contrast, late-onset, or sporadic, AD accounts for the majority of cases, but the causes of this AD type are less defined and multi-factorial. The multitude and complexity of risk factors that contribute to the development of sporadic AD have created a puzzle for researchers and clinicians and creates a barrier to effective treatment.

## Risk factors

The second type of AD is called late-onset AD. While no specific genes have been determined to directly cause late-onset AD (LOAD), some genes and gene variants have been identified as increasing the risk for developing LOAD, such as Apolipoprotein E (APOE). APOE is an important lipid transport protein (Mahley, 1988; Mahley and Rall, 2000). Single-nucleotide polymorphisms in the APOE gene on chromosome 19 result in three major isoforms: APOE2, E3, and E4 (Rall et al., 1982; Weisgraber et al., 1982; Laws et al., 2003; Seripa et al., 2011). The E3 isoform is most common in the human population and neither decreases nor increases risk for developing AD (Martins et al., 1995; Corbo and Scacchi, 1999). A carrier with at least one copy of the APOE4 allele has a higher risk of developing AD and may develop it at an earlier age (Corder et al., 1993; Mayeux et al., 1993; Saunders et al., 1993; Strittmatter et al., 1993; Grossman et al., 2010). Further, the number of APOE4 alleles carried (one versus two) is proportional to the risk of AD (Corder et al., 1993; Farrer et al., 1997). Individuals with two copies of the E4 allele are about fifteen times more likely to develop AD than those with the E3/E3 genotype (Farrer et al., 1997; Liu et al., 2013).

Of course, age is the greatest risk factor for developing AD (Gao et al., 1998; Alzheimer’s Association, 2019b), but dementia is not an inevitable consequence of aging. Increased risk for LOAD also comes from environmental and biological factors. For example, biological sex can confer risk for AD; about two-thirds of AD patients are women (Alzheimer’s Association, 2014). Researchers consistently observe that women are more likely to be diagnosed with AD than men (Gao et al., 1998; Vest and Pike, 2013; Mielke et al., 2014; Pike, 2017; Ferretti et al., 2018; Mielke, 2018), but the reasons for the discrepancy are largely unknown. One hypothesis for women’s increased risk of AD is the loss of endogenous estrogens (i.e., 17-β estradiol) during menopause (Bove et al., 2014; Mosconi et al., 2021) because estrogen is known to be important for memory and cognition (Su et al., 2012; Rettberg et al., 2014; Hampson, 2018).

Medical conditions and lifestyle factors can also influence the risk for AD. For example, metabolic conditions such as Metabolic Syndrome (Razay et al., 2007; Campos-Peña et al., 2017), obesity (Christensen and Pike, 2017; Jones and Rebeck, 2018), diabetes (Luchsinger, 2008; Pugazhenthi et al., 2017), and insulin resistance (Craft, 2009) can increase the risk of developing AD. Vascular conditions such as hypertension (Craft, 2009), stroke (Breteler, 2000; de la Torre, 2002), and cardiac disease (O’Brien and Markus, 2014) contribute to increased AD risk, as well. Finally, mental health conditions and stress contribute to one’s likelihood of being diagnosed with AD. Depression and anxiety have been repeatedly linked to AD risk (Ownby et al., 2006; Rasmussen et al., 2018; Cantón-Habas et al., 2020), and these diagnoses may even be associated with an earlier age of AD onset (Eijansantos et al., 2021). Chronic stress can also have detrimental effects on the brain that increases the risk of developing AD (Bisht et al., 2018; Justice, 2018), such as inflammation and altered glucose metabolism (Machado et al., 2014). The variety and complexity of AD risk factors is troubling, but a better understanding of the relationship between risk factors and the disease pathology could lead to new precision-based treatment and prevention options.

## Disease pathology

### Amyloid beta pathogenesis

For decades, the “Amyloid cascade hypothesis” of AD has been the prominent mechanistic theory of disease development (Hardy and Allsop, 1991; Hardy and Higgins, 1992). While there is some evidence to suggest otherwise (Kametani and Hasegawa, 2018), it is well known that the buildup of amyloid particles strongly correlates with AD pathology (Glenner and Wong, 2012; Selkoe and Hardy, 2016). Aβ is a 39–47 residue protein created by the processing and cleavage of APP (Chen et al., 2017). In non-amyloidogenic processing, APP is cleaved by ADAM family proteases to yield soluble APP, which is released into the extracellular space (Hampel et al., 2021). Amyloidogenic processing of APP by BACE1 β-secretase and γ-secretase, composed of Presenilin 1 or 2, results in the formation of a distinct form of soluble APP, as well as Aβ monomer, for extracellular release (LaFerla et al., 2007; Hampel et al., 2021). Aβ monomers can aggregate into various forms, such as oligomers, which are soluble and can spread throughout the brain, and fibrils, which are insoluble, larger in size, and are prone to forming amyloid plaques (Chen et al., 2017). Aβ is thought to have physiological roles in the healthy brain, including responding to neuronal challenges and injury by repairing leaks in the blood-brain barrier, for example (Brothers et al., 2018). However, extracellular Aβ can become toxic when it interferes with synaptic receptors (Westmark, 2013), is internalized and aggregates to trigger cell death (Friedrich et al., 2010), and when plaque formation and deposition occurs, as is observed in AD (Hardy and Allsop, 1991; Seeman and Seeman, 2011).

While amyloid beta has been a significant topic of study in AD and the primary treatment target, clinical trials to target Aβ have not been successful. Immunotherapies directed at Aβ in AD patients can be effective in decreasing Aβ deposition in the brain, but it has not led to an improvement in clinical symptoms or decreased accumulation of Tau (Ostrowitzki et al., 2012; Doody et al., 2014; Salloway et al., 2014). In addition, the actual level and aggregation of Aβ can vary among AD patients. For example, some patients may exhibit very few Amyloid deposits, and some cognitively normal patients may have more Amyloid deposits in their brain (Edison et al., 2007; Li et al., 2008). Therefore, it may be prudent to turn to Tau as a predictor and potential treatment target in AD. Positron emission tomography (PET) studies have shown the spatial patterns of Tau tracer binding are closely linked to the patterns of neurodegeneration and the clinical presentation in AD patients (Bejanin et al., 2017; Okamura and Yanai, 2017).

### Tau pathology and neurodegeneration

Tau promotes the assembly and stability of microtubules and other microtubule-associated proteins (Barbier et al., 2019). Tau also helps regulate microtubule functions, such as neurite outgrowth and axonal transport (Johnson and Stoothoff, 2004), by binding to microtubules *via* its highly conserved microtubule-binding repeat domains R1-R4 (Goedert et al., 1988). Microtubules fluctuate between a stable state and dynamic instability, and a balance between these two forms is necessary to ensure proper neuronal communication and survival (Dubey et al., 2015). Tau phosphorylation is an important part of this system, as this physiological phenomenon decreases the affinity of Tau to microtubules and maintains their dynamic nature to ensure proper neuron function (Lindwall and Cole, 1984; Mandelkow et al., 1995). However, abnormal, or hyper-, phosphorylation of Tau decreases the stability of microtubules, resulting in increased neurite branching, reduced axonal transport, and synapse retraction (Jameson et al., 1980; Dubey et al., 2015). Tau hyperphosphorylation, and the subsequent destabilization of microtubules, is observed in neurodegenerative diseases such as Parkinson’s disease, Amyotrophic Lateral Sclerosis (ALS), and Alzheimer’s disease (Iqbal et al., 1986; Alonso et al., 1996; Dubey et al., 2015). Indeed, enhancing microtubule stability in a mouse model of AD rescues Amyloid and Tau pathology as well as cognitive deficits (Fernandez-Valenzuela et al., 2020).

Hyperphosphorylated Tau in the AD brain can aggregate into oligomers, paired helical filaments, and ultimately neurofibrillary tangles (Goedert et al., 1988; Bancher et al., 1989; Šimić et al., 2016; Jouanne et al., 2017; Hill et al., 2020). Once aggregated, Tau is more difficult to de-phosphorylate by phosphatases (Miao et al., 2019). Oligomeric Tau can then act as a “seed” and cause other Tau molecules in nearby neurons to aggregate into fibrils (Medina and Avila, 2014; Shafiei et al., 2017; Hill et al., 2020). Tau oligomers have been found to be the specific source of deficits in axonal transport in neurons (Ward et al., 2012; Shafiei et al., 2017; Hill et al., 2020), which can lead to neuronal death. Researchers H. Braak and E. Braak proposed a staging of neuropathological Tau deposition in the brain through immunohistochemical staining (Braak and Braak, 1991), which has been optimized over time to allow pathologists to more consistently judge the stage of Tau accumulation and whether AD should be diagnosed in a patient post-mortem (Braak et al., 2006).

The neurodegeneration (i.e., neuronal loss) and brain atrophy observed in AD aligns well with the formation and staging of Tau in neurofibrillary tangles (Braak and Braak, 1991; Gómez-Isla et al., 1997; Smith, 2002). The first regions affected in the AD brain are the entorhinal cortex and hippocampus, followed by parts of the neocortex and temporal lobe, at which point mild cognitive impairment (MCI) may be apparent in patients. Finally, the pathology extends to the occipital lobe and then frontal areas of the cortex (Braak and Braak, 1991; Scahill et al., 2002; Smith, 2002), leading to late-stage personality changes and difficulties with everyday tasks. The atrophy of these regions is paired with enlargement of the ventricles (Nestor et al., 2008; Apostolova et al., 2012). Neuronal loss is thought to be the main pathological catalyst for cortical atrophy and ventricular enlargement (Serrano-Pozo et al., 2011).

### Mechanisms of cell death

There are multiple potential mechanisms of neuronal death in AD pathogenesis. First, there is increased evidence of apoptosis in the AD brain (Mattson, 2000). Apoptosis is a tightly regulated form of programmed cell death that can be induced by a variety of factors in AD, including endoplasmic reticulum stress, increased levels of intracellular calcium, oxidative stress, DNA damage, and the presence of pathological Aβ (Behl, 2000). Apoptosis relies on caspases – cysteine proteases that cleave a range of cellular substrates (Li and Yuan, 2008). Evidence of caspase 3 and caspase 8 activation have been observed in AD (Stadelmann et al., 1999; Rohn et al., 2001a; Su et al., 2001); their activation has also been linked with neurofibrillary tangle formation (Rohn et al., 2001b,^2002^). Caspases likely cleave Tau early in AD development, which could precede its hyperphosphorylation (Rissman et al., 2004). Additionally, caspase 3 is involved in the cleavage of APP (Gervais et al., 1999), so it could contribute to Amyloid pathology as well.

As discussed previously, the balance between microtubule stability and dynamics is important for axonal transport, conserving neuronal morphology and polarity, and regulating signaling cascades (Dent et al., 2003; Yu et al., 2008; Dubey et al., 2015). Dynamic microtubules modulate spine density and synaptic plasticity (Jaworski et al., 2009; Fanara et al., 2010). The loss of dendritic spines and subsequent deficits in long-term potentiation seem to be one of the earliest changes in neurodegeneration, and this process could be regulated through NMDARs (Tackenberg and Brandt, 2009), therefore the maintenance between stable and dynamic microtubules is critical. In healthy neurons, Tau binds to microtubules to promote their polymerization and stability (Weingarten et al., 1975; Cleveland et al., 1977; Panda et al., 2003). As stated previously, exaggerated microtubule instability is characteristic of neurodegenerative disorders such as AD (Iqbal et al., 1986; Alonso et al., 1996; Dubey et al., 2015). While the importance of microtubule function is undoubted, it is still unclear if microtubule instability is a direct cause of cell death or if it is a consequence of neurodegenerative pathways that involve Tau, Aβ, or other factors.

Finally, DNA damage is a well-accepted trigger of cell death and apoptosis in AD (Lin et al., 2020). Single-strand and double-strand breaks (DSBs) of DNA are found to be elevated in the hippocampus from AD patients (Adamec et al., 1999). A large contributor to DNA damage in AD is oxidative stress, i.e., the increased presence of oxygen-derived radicals (Lyras et al., 1997; Gabbita et al., 1998). Oxidative stress is thought to be an early event in AD neurodegeneration (Bradley-Whitman et al., 2014; Shanbhag et al., 2019). In addition to DNA damage, deficiencies in DNA repair further compromise cellular health in AD. Deficiencies in both types of DSB repair, homologous recombination (HR) and non-homologous end-joining (NHEJ), are implicated in AD. Because neurons in an aged brain are mostly post-mitotic, and the high transcriptional activity in mature neurons is a major source of DSBs that are repaired *via* NHEJ (Crowe et al., 2006; Suberbielle et al., 2013; Madabhushi et al., 2015; Ceccaldi et al., 2016), it seems logical that deficiencies in NHEJ would be the main source of DSB accumulation in AD. However, evidence of HR deficiencies were also found in human patients and AD mouse models, such as decreased ATM expression (Shen et al., 2016) and less RAD51-positive foci in cells (Yu et al., 2018). Overall, DNA damage and insufficient DNA repair certainly contribute to cell death in AD, and researchers are working to determine the precise mechanisms by which this occurs.

## DEK nuclear functions

Recently, there has been growing attention toward the DEK protein and its role in brain health and disease. DEK is a phosphoprotein which regulates many nuclear processes, such as chromatin remodeling, transcription, and DNA replication and repair. Early in its discovery, DEK was linked with protection against mutagens and radiation in fibroblasts deficient in ATM (Meyn et al., 1993), a crucial protein kinase for homologous recombination DNA double strand break repair. Subsequent experiments identified that DEK binds directly to DNA, with a preference for supercoiled DNA (Waldmann et al., 2002, 2003), and that its interaction with DNA at specific sites positions DEK to regulate transcription and signal transduction (Fu et al., 1997). *In vivo*, the majority of DEK within a cell is associated with chromatin (Kappes et al., 2001), where it alters the superhelical density of DNA (Alexiadis et al., 2000) and is crucial for maintaining chromatin architecture, such as telomeres (Ivanauskiene et al., 2014) and the balance between heterochromatin and euchromatin (Kappes et al., 2011; Sandén et al., 2014; Waidmann et al., 2014). The function of DEK as a chromatin architectural protein is very similar to that of high-mobility group (HMG) proteins (Waldmann et al., 2004).

Through its functions of binding DNA and regulating chromatin structure, it is suggested that DEK can alter gene transcription. DEK has been identified as both a transcription coactivator and repressor through interactions with transcription factors such as AP-2α on the APOE promoter (Campillos et al., 2003), C/EBPα in myeloid cells (Koleva et al., 2012), and Daxx (Hollenbach et al., 2002). DEK may also serve as a histone chaperone to act as a transcriptional coactivator for nuclear receptors (Sawatsubashi et al., 2010) and to inhibit transcription and acetyltransferase activity of p300 and PCAF (Ko et al., 2006). The loss of DEK results in altered expression of transcriptional regulators and known DEK-bound genes (Shibata et al., 2010; Sandén et al., 2014). DEK mainly binds to genes that are ubiquitously expressed throughout various tissues in the body (Sandén et al., 2014), suggesting that DEK has a widespread role in gene expression regulation.

DEK regulates cellular health, as demonstrated by the stimulation of cellular proliferation *via* the Wnt/β-catenin pathway with enhanced DEK expression (Privette Vinnedge et al., 2011, 2015; Yang et al., 2020). DEK expression modulates the upregulation of cell cycle and DNA replication-associated genes (Nakashima et al., 2017). The interaction of DEK with DNA may also contribute to its facilitation of cellular proliferation, as DEK promotes proliferation under conditions of DNA replication stress by advancing replication fork progression (Deutzmann et al., 2015). Loss of DEK expression compromises cellular health by triggering deficiencies in DNA damage repair. DEK is important for DSB repair by both NHEJ (Kavanaugh et al., 2011) and HR (Smith et al., 2017), and DEK loss results in increased levels of exaggerated cellular responses to genotoxic stress (Kavanaugh et al., 2011). The presence of DEK also mediates anti-apoptotic mechanisms, likely through interference with p53 (Wise-Draper et al., 2006), thereby inhibiting cell death in many cases (Wise-Draper et al., 2005; Khodadoust et al., 2009). Interestingly, the overexpression of DEK was also reported to cause apoptosis through caspases 9 and 3 in the Drosophila eye (Lee et al., 2008). Therefore, it is likely that DEK expression is tightly regulated to maintain healthy cellular function, because the loss of DEK, or its overexpression, is deleterious and may cause cell death.

DEK expression is primarily regulated at the transcriptional level and is a target gene of many pro-proliferative transcription factors including ERα, NF-Y, YY1, and E2F (Sitwala et al., 2002; Carro et al., 2006; Privette Vinnedge et al., 2012). However, regulation of post-transcriptional DEK expression levels is even less well understood. The FBXW7 and SPOP E3 ubiquitin ligases are the only known molecular mechanisms that control DEK protein levels post-transcriptionally, both of which have only been noted in solid tumors (Babaei-Jadidi et al., 2011; Theurillat et al., 2014). Finally, post-translational modifications, including phosphorylation and ADP-ribosylation, are known to control DEK functions such as DNA binding and dimerization (Kappes et al., 2004, 2008; Fahrer et al., 2010; Privette Vinnedge et al., 2013). However, the functional consequences of these DEK modifications for disease progression remain undefined. Finally, no significant mutations in the *DEK* gene have been identified in any clinical populations to date, suggesting that DEK expression, mis-localization, and post-translational modifications are the driving mechanisms by which DEK is associated with diseases such as cancer, neurodegeneration, and auto-immune disorders.

## DEK and diseases in the periphery

DEK was initially discovered as a fusion protein with a nucleoporin (DEK-CAN, a.k.a.: DEK-NUP214) in a subset of acute myeloid leukemia (AML) patients, and indeed, DEK does play a role in disorders of white blood cell function, including autoimmune disorders and malignancies like AML (Garcon et al., 2005). Anti-DEK antibodies have been found in various inflammatory autoimmune conditions, such as sarcoidosis, systemic sclerosis, rheumatoid arthritis (Sierakowska et al., 1993; Dong et al., 2000), systemic lupus erythematosus (Wichmann et al., 2000), and juvenile idiopathic arthritis (Mor-Vaknin et al., 2011). The interaction between DEK and DEK autoantibodies can induce inflammation and interrupting this interaction can alleviate this detrimental immune response (Mor-Vaknin et al., 2017). DEK’s role in inflammation even extends beyond this class of disorders. Recently, it was determined that increased DEK expression in breast cancer cells results in differential expression of multiple cytokines that contribute to M2 polarization of tumor-associated macrophages, potentially creating an immune suppressed tumor microenvironment (Pease et al., 2020).

The overexpression of DEK is found in a majority of solid tumors, including retinoblastoma (Grasemann et al., 2005), adenocarcinoma (Piao et al., 2014), lung cancer (Shibata et al., 2010; Wang et al., 2014; Yang et al., 2020), bladder cancer (Carro et al., 2006; Datta et al., 2011), prostate cancer (Lin et al., 2015), cervical cancer (Wu et al., 2008), melanoma (Khodadoust et al., 2009), breast cancer (Liu et al., 2012; Privette Vinnedge et al., 2015), and esophageal (Yi et al., 2015) and oral squamous cell carcinoma (Nakashima et al., 2017). High DEK expression leads to tumorigenic phenotypes by inhibiting apoptosis and cellular senescence, meanwhile promoting proliferation and cellular transformation (Wise-Draper et al., 2009; Riveiro-Falkenbach and Soengas, 2010). It is important to note that DEK overexpression on its own *in vivo* is not tumorigenic but requires initiating mutations through chemical carcinogenesis or cooperating tumor initiating mutations (Matrka et al., 2018). Therefore, DEK overexpression in cancer is tumor-promoting, not tumor initiating. The first foray to studying DEK in the brain was through DEK’s association with brain tumors of glial origin (Feng et al., 2017). DEK was found to be highly expressed in astrocytic tumors, and DEK silencing in glioblastoma cells *in vitro* inhibited cell proliferation and induced apoptosis. This suggests that there is a convergence in the findings of DEK’s potential roles in peripheral tissues and the central nervous system.

## DEK in the brain

The discovery of the DEK gene and protein is relatively recent, and even more so is the study of DEK expression and function in the brain. Our group first characterized DEK expression in the murine brain in 2018 to find that DEK is expressed throughout the brain in both adult males and females, including in regions important for learning and memory (Ghisays et al., 2018). Interestingly, female mice had higher DEK expression in the hippocampal CA1 region than males, but there were no sex differences in any of the other brain regions assessed (Ghisays et al., 2018). While interesting, we cannot determine if this sex difference in CA1 DEK expression is biologically relevant, or if it is due to the organizational or activational effects of gonadal hormones, since randomly cycling female mice were used for the immunohistochemical analyses in this paper and the expression of markers for synaptic plasticity in the CA1 is sensitive to gonadal hormones (Ghisays et al., 2018). However, in this paper, the variability in cell counts for DEK and other markers was not limited to sex, suggesting that potential variability in DEK expression between males and females is not due solely to the activational effects of gonadal hormones (Ghisays et al., 2018). Furthermore, this sex difference in DEK expression may be explained by DEK being an estrogen receptor alpha (ERα) target gene (Privette Vinnedge et al., 2012) and that, among all hippocampal regions, the CA1 has the highest expression of ERα (Mitterling et al., 2010). Therefore, it is possible that higher DEK expression in the CA1 region of the female murine hippocampus may be due to the higher expression levels of ERα in this brain region. It will be important to uncover DEK’s specific functions in the brain to fully understand its role in the female vs. male brain.

DEK is expressed in multiple neural cell types in the mouse brain: neurons, microglia, and astrocytes (Ghisays et al., 2018). We confirmed these findings in humans, using data from online tools such as Brain RNA-Seq and Human Protein Atlas. DEK is expressed in most major cell types, including astrocytes, microglia, oligodendrocytes, endothelial cells, and neurons (Greene et al., 2022). DEK expression is approximately two to three-fold higher in fetal astrocytes than other cell types analyzed, followed by relatively high expression levels in microglia and oligodendrocytes (Greene et al., 2022). DEK expression levels are similar in neurons, mature astrocytes, and endothelial cells (Greene et al., 2022). In addition, DEK is expressed in the human forebrain, midbrain, and hindbrain; the areas with the highest *DEK* RNA expression are the cerebellum, thalamus, and cerebral cortex (Greene et al., 2022). Further analysis of DEK expression in the healthy human brain revealed that DEK is most highly expressed during fetal development in the forebrain and its expression rapidly declines after birth to remain steady throughout life (Greene et al., 2022).

The regulation of DEK expression has broad functional implications throughout the brain. Our recent pathway analyses found that genes whose expression is correlated with that of DEK are related to neurotransmission and synaptic signaling, inflammatory responses, glutamate receptor signaling, neuron death, and hormone regulation. Pathways related to DEK’s known functions in the periphery are also reflected in DEK-related functions in the brain: regulation of the cell cycle, DNA repair and replication, RNA processing, and metabolism (Greene et al., 2022). When DEK-associated pathways were separated by age group, one pathway that was uniquely related to DEK expression during adulthood (20 + years old) was superoxide metabolism, a function important in aging (Greene et al., 2022). The fact that there are differences in DEK-associated biological functions across age groups suggests that DEK could play different roles in the brain throughout a person’s lifetime.

### DEK and neurodegeneration

DEK was first associated with age-related disorders in 2018, when we identified Alzheimer’s disease, dementia, senile plaques, amyloid plaques, vascular dementia, and age-related cognitive decline as potential DEK loss-associated diseases using RNA-Seq analyses (Ghisays et al., 2018). A further look into DEK expression in humans revealed that, while there was no difference in DEK expression between males and females in the non-demented brain (O’Donovan et al., 2018), DEK expression was lower in women with dementia than in men (O’Donovan et al., 2018). This suggests that there may be a sex-specific role of DEK in neurodegeneration. Alzheimer’s disease (AD) disproportionately affects more women than men, therefore it is worth exploring the role of DEK in this common, debilitating disorder. Given that at normal levels, DEK functions to promote cellular health, we postulate that nuclear DEK could be neuroprotective and that the loss of DEK is related to dementia and AD.

Publications from our group and others have supported this hypothesis. In 2020, we reported that DEK loss in SH-SY5Y cells, isolated from a female patient and differentiated to neurons, resulted in AD phenotypes, such as cell death and Tau pathology (Greene et al., 2020). Specifically, the loss of DEK expression *via* shRNA lentivirus induced cell death *via* apoptosis, impaired neurite formation in neuronal cells, and increased Tau phosphorylation at sites known to hinder Tau binding to microtubules (Greene et al., 2020). As mentioned earlier, Tau hyperphosphorylation and the subsequent destabilization of microtubules is observed in neurodegenerative diseases such as Alzheimer’s. We also observed decreased levels of β-catenin after DEK loss (Wu et al., 2008), therefore it is possible that GSK3β, a kinase in the Wnt/β-catenin pathway, could be deregulated with DEK loss. GSK3β is a kinase known to target Tau for phosphorylation in AD (Wagner et al., 1996; Hanger et al., 2009).

Notably, Tau phosphorylation is not the only mechanism by which Tau aggregates and spreads throughout the brain in AD. Acetylation of Tau also likely contributes to Tau pathology by targeting soluble Tau and altering it to result in toxic oligomers which contribute to the reduced solubility of Tau and its aggregation (Cohen et al., 2011; Irwin et al., 2012; Lucke-Wold et al., 2017). A histone acetyltransferase, p300, is known to be involved in Tau acetylation and the inhibition of p300 specifically reduced pathological Tau in primary neurons (Min et al., 2010). Interestingly, researchers found that DEK exerts an inhibitory effect on p300-mediated histone acetyltransferase activity (Ko et al., 2006). Therefore, multiple mechanisms of Tau pathology should be investigated in relation to DEK to gain a better understanding of DEK’s role in dementia and AD.

Other recent studies have also identified DEK as a potential target in neurodegenerative diseases. Miao, et al. found that the overexpression of miR-138, a key microRNA known to promote Tau phosphorylation, downregulated DEK expression in SH-SY5Y cells, resulting in the inactivation of AKT and elevated expression levels of proapoptotic caspase-3 (Miao et al., 2020). DEK was among a small group of genes found to be differentially expressed between pre-symptomatic and symptomatic Huntington’s disease (Christodoulou et al., 2020). DEK was also identified as a hub gene in the comparison of differentially expressed genes between Huntington’s disease patients and healthy controls (Xiang et al., 2020). Finally, data from a single-cell transcriptomic analysis of AD patients found that DEK expression in excitatory neurons is lower in patients with early stages of AD relative to healthy controls (Mathys et al., 2019). Multiple research groups have now suggested that decreased or dysregulated DEK expression could be a driver of neurodegenerative diseases like Alzheimer’s and Huntington’s.

## Future directions

There is increasing evidence to suggest that DEK expression is protective in neuronal cells and that the regulation of DEK expression could be important in the context of dementia. However, there are many knowledge gaps to address in future studies. First, we do not know how DEK loss could impact neurodegeneration differently in males and females. We observed sex differences in DEK expression in the demented human brain but not in the healthy brain (O’Donovan et al., 2018; Greene et al., 2022). DEK is known to be a target gene of sex hormone receptors (Privette Vinnedge et al., 2012), and hormone regulation and signaling have been identified as DEK-associated pathways in the human brain (Greene et al., 2022). Therefore, it will be important to consider the potential sex-specific effect of DEK loss in AD and other age-related dementias. Further, other AD risk factors, such as APOE genotype and metabolic conditions, should be studied in relation to neural DEK to better understand what biological and environmental risk factors could cause decreased DEK expression in the brain, leading to AD phenotypes.

To date, *in vitro* studies of DEK expression in AD have focused on neuronal cell models. Because the pathophysiology of AD involves cell types beyond neurons, such as microglia and astrocytes, it will be imperative to study the consequences of DEK loss in other major neural cell types. Chronic activation of glial cells is consistently observed in the AD brain accompanying the pathological aggregation of Aβ and Tau, which can lead to neuronal death by production of reactive oxygen species (ROS), phagocytosis of neurons by activated microglia, or secretion of neurotoxic factors by reactive astrocytes (Shi and Holtzman, 2018). Astrocytes also play a key role in glutamate processing and homeostasis, which can go awry in AD (Andersen et al., 2021). A previous study found that extracellular levels of glutamate were decreased in DEK-overexpressing cells (Matrka et al., 2017), therefore, it is possible that altered DEK expression could affect glutamate levels and processing. Future studies will be required to determine if altered DEK expression in glial cells could impact AD pathology.

Much of our work studying the effects of DEK loss on phenotypes of AD to date have focused on Tau pathology. However, to understand more of the full picture of how DEK expression may be related to AD, we must evaluate the other pathological hallmark of this disease, i.e., amyloidogenic processing of APP and the presence of Aβ plaques. Miao et al. found that in Aβ-treated SH-SY5Y cells, miR-138 expression was increased and DEK expression decreased, which resulted in indications of apoptosis (Miao et al., 2020). When DEK expression was rescued in these Aβ-treated cells, AKT was activated and cleaved caspase 3 expression was reduced (Miao et al., 2020). While interesting, these results don’t offer insights into the specific effects of Aβ expression and pathology on DEK expression and vice versa.

**FIGURE 1:**
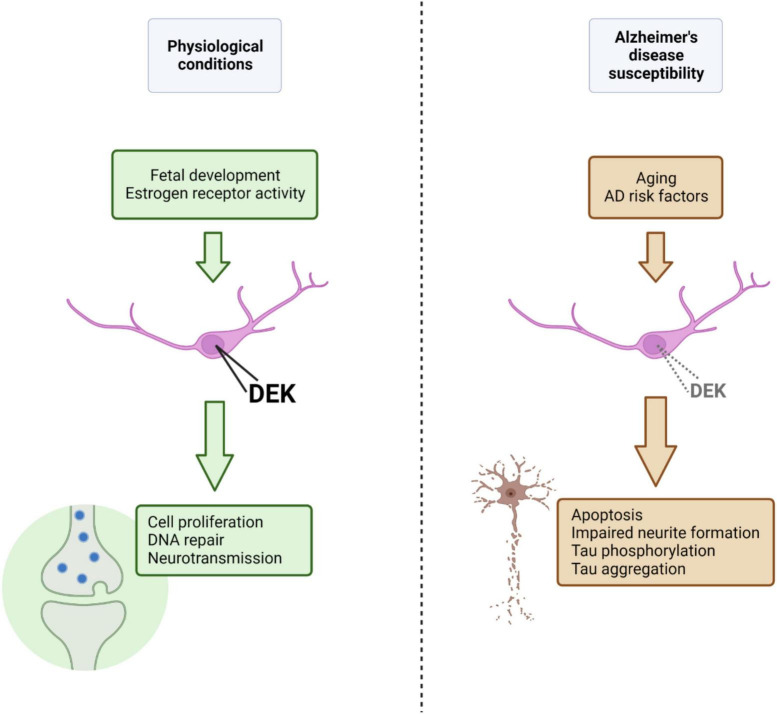
Under physiological conditions, nuclear DEK expression, promoted and maintained by factors such as estrogen receptor activity or age (i.e., fetal development), is important for a variety of processes in the central nervous system (represented in green). Aging and other AD risk factors, in theory, could lead to decreased nuclear DEK expression (although the effect of specific AD risk factors on DEK expression is largely unknown), resulting in aberrant cellular processes, declined neuronal health, and phenotypes of AD (represented in brown). Figure created using BioRender.com.

Overall, there is a small, but growing, body of research to suggest that DEK is associated with neurodegenerative conditions such as Alzheimer’s and Huntington’s disease. The inverse in transcriptional regulation of genes between AD and cancer (Lanni et al., 2021) gives more credence to the notion that DEK loss could be related to AD, given DEK’s predominant role as an oncogene when overexpressed in peripheral tissues. It is likely that DEK expression is fine-tuned to maintain normal physiological functions and that overexpression or loss of DEK expression may have detrimental consequences for cellular health. New knowledge about DEK expression and function in the healthy brain will also help us understand potential roles DEK could have in the aged brain and how dysregulated DEK expression could be a driver of neurodegeneration. For a summary of the findings and theoretical mechanisms discussed here linking DEK loss with AD, please refer to Figure 1. Future studies should investigate sex-specific effects of DEK loss, the relationship between DEK and Aβ, the association between AD risk factors and DEK expression, and the effect of DEK loss in specific brain regions and cell types on phenotypes of dementia.

## Author contributions

LP and MS provided guidance on content, editing, and funding. AG wrote the manuscript and advised on content. All authors contributed to the article and approved the submitted version.

## Abbreviations

Aβ: amyloid beta
AD: Alzheimer’s disease
ADAM: A disintegrin and metalloproteases
AKT: protein kinase B
AML: acute myeloid leukemia
AP-2α: activator protein 2α
APOE: Apolipoprotein E
APP: amyloid precursor protein
ATM: ataxia-telangiectasia mutated
BACE1: Beta-secretase 1
C/EBP: CCAAT-enhancer-binding protein
Dek: Murine Dek gene/protein
DEK: Human DEK gene/protein
DSB: double-strand break
GSK3β: Glycogen synthase kinase 3 beta
HR: homologous recombination
LOAD: late-onset Alzheimer’s disease
MCI: mild cognitive impairment
miRNA: micro RNA
NHEJ: non-homologous end joining
NMDAR: N-methyl -d-aspartate receptor
PET: positron emission tomography
shRNA: short hairpin RNA
Wnt: wingless-related integration site.
